# Investigations on the Degradation of the Bile Salt Cholate via the 9,10-*Seco*-Pathway Reveals the Formation of a Novel Recalcitrant Steroid Compound by a Side Reaction in *Sphingobium* sp. Strain Chol11

**DOI:** 10.3390/microorganisms9102146

**Published:** 2021-10-14

**Authors:** Franziska Maria Feller, Sebastian Eilebrecht, Ruslan Nedielkov, Onur Yücel, Julia Alvincz, Gabriela Salinas, Kevin Christopher Ludwig, Heiko Möller, Bodo Philipp

**Affiliations:** 1 Institute for Molecular Microbiology and Biotechnology, University of Münster, Corrensstr. 3, 48149 Münster, Germany; franziska.feller@uni-muenster.de (F.M.F.); onuryuecel@googlemail.com (O.Y.); kevin.ludwig@uni-bonn.de (K.C.L.); 2 Fraunhofer Attract Eco’n’OMICs, Fraunhofer Institute for Molecular Biology and Applied Ecology IME, Auf dem Aberg 1, 57392 Schmallenberg, Germany; sebastian.eilebrecht@ime.fraunhofer.de (S.E.); julia.alvincz@ime.fraunhofer.de (J.A.); 3 Institute for Chemistry, University of Potsdam, Karl-Liebknecht-Straße 24-25, 14476 Potsdam, Germany; ruslan.nedielkov@uni-potsdam.de (R.N.); heiko.moeller@uni-potsdam.de (H.M.); 4 NGS-Services for Integrative Genomics, Institute for Human Genetics, University of Göttingen, 37077 Göttingen, Germany; gsalina@gwdg.de; 5 Department for Environmental Microbiology, Fraunhofer Institute for Molecular Biology and Applied Ecology IME, Auf dem Aberg 1, 57392 Schmallenberg, Germany

**Keywords:** bile salts, steroid degradation, bacterial metabolism, side reaction, steroid compound

## Abstract

Bile salts such as cholate are steroid compounds from the digestive tracts of vertebrates, which enter the environment upon excretion, e.g., in manure. Environmental bacteria degrade bile salts aerobically via two pathway variants involving intermediates with Δ^1,4^- or Δ^4,6^-3-keto-structures of the steroid skeleton. Recent studies indicated that degradation of bile salts via Δ^4,6^-3-keto intermediates in *Sphingobium* sp. strain Chol11 proceeds via 9,10*-seco* cleavage of the steroid skeleton. For further elucidation, the presumptive product of this cleavage, 3,12β-dihydroxy-9,10-*seco*-androsta-1,3,5(10),6-tetraene-9,17-dione (DHSATD), was provided to strain Chol11 in a co-culture approach with *Pseudomonas stutzeri* Chol1 and as purified substrate. Strain Chol11 converted DHSATD to the so far unknown compound 4-methyl-3-deoxy-1,9,12-trihydroxyestra-1,3,5(10)7-tetraene-6,17-dione (MDTETD), presumably in a side reaction involving an unusual ring closure. MDTETD was neither degraded by strains Chol1 and Chol11 nor in enrichment cultures. Functional transcriptome profiling of zebrafish embryos after exposure to MDTETD identified a significant overrepresentation of genes linked to hormone responses. In both pathway variants, steroid degradation intermediates transiently accumulate in supernatants of laboratory cultures. Soil slurry experiments indicated that bacteria using both pathway variants were active and also released their respective intermediates into the environment. This instance could enable the formation of recalcitrant steroid metabolites by interspecies cross-feeding in agricultural soils.

## 1. Introduction

Bile salts are amphiphilic steroids, which are mainly synthesized in the liver of vertebrates and have diverse functions in digestion and signaling [1,2]. Mammalian bile salts carry a C_5_-carboxylic side chain attached to C-17 of their steroid skeleton and differ in the number, position, and stereochemistry of hydroxyl groups. A total of 95% of bile salts are recycled in the enterohepatic cycle, but a certain amount is not reabsorbed and therefore excreted with feces and urine, e.g., 400–600 mg bile salts per day and human [3].

**Figure 1:**
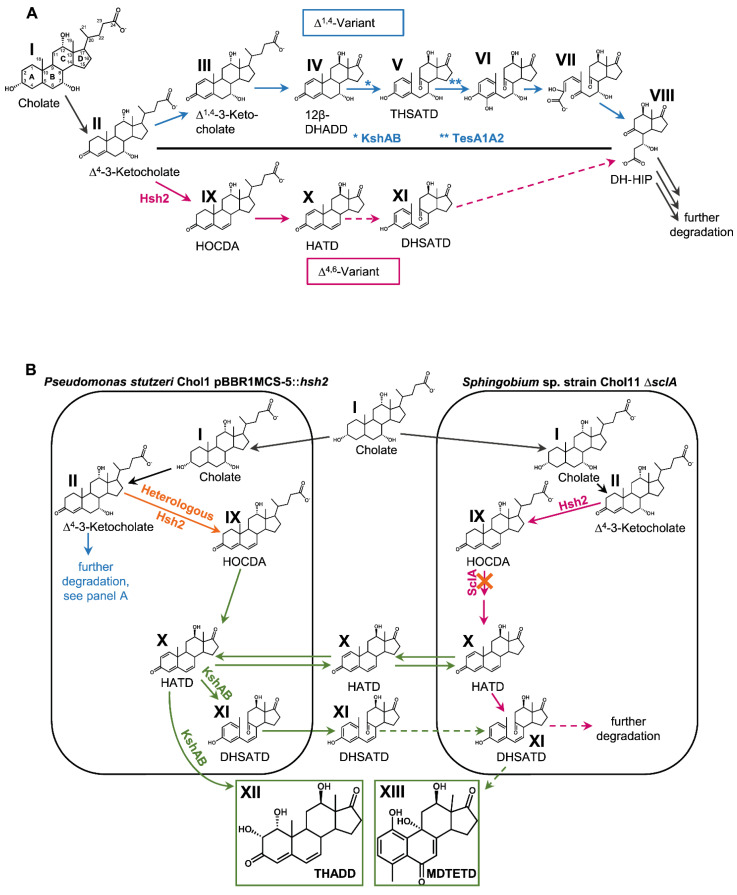
(**A**) Section of cholate degradation via the Δ^1,4^- and Δ^4,6^-variants of the 9,10-*seco*-pathway. For a detailed description and illustration of the pathway, including all known enzymes involved in bile salt degradation, see review [6]. (**B**) Principle of the co-culture designed for providing DHSATD (XI) to *Sphingobium* sp. strain Chol11. The heterologous expression of Hsh2 in *P. stutzeri* Chol1 leads to the accumulation of dead-end intermediates DHSATD (XI) and THADD (XII). The *sclA* deletion mutant strain Chol11 Δ*sclA* lacking the steroid C_5_-CoA ligase can use cholate only very slowly. Intermediates are produced inside the cells but can be found in the culture supernatant, most probably due to efflux. Black arrows: reaction present in both pathways, blue arrows: degradation via Δ^1,4^-pathway, red arrows: degradation via Δ^4,6^-pathway, orange arrows: changes in metabolism compared to wild types, green arrows: cross-feeding reaction, solid lines: known reactions, dotted lines: reactions found in this study. I: Cholate, II: ∆^4^-3-Ketocholate, III: ∆^1,4^-3-Ketocholate, IV: 12β-DHADD (7α,12β-Dihydroxy-androsta-1,4-diene-3,17-dione), V: THSATD (3,7,12-Trihydroxy-9,10-*seco*-androsta-1,3,5(10)-triene-9,17-dione), VI: 3,4,7,12-Tetrahydroxy-9,10-*seco*-androsta-1,3-5(10)-triene-9,17-dione, VII: 4,5-9,10-Di*seco*-3,7,12-trihydroxy,4,9,17-trioxoandrosta-1(10)2-diene-4-oate, VIII: DH-HIP (3,7β-Dihydroxy-*H*-methyl-hexahydro-indanone-propanoate), IX: HOCDA (12α-Hydroxy-3-oxo-4,6-choldienoate), X: HATD (12-Hydroxy-androsta-1,4,6-triene-3,17-dione), XI: DHSATD (3,12β-Dihydroxy-9,10-*seco*-androsta-1,3,5(10),6-tetraene-9,17-dione), XII: THADD (1α,2α,12β-Trihydroxy-androsta-4,6-triene-3,17-dione), XIII: MDTETD (4-Methyl-3-deoxy-1,9,12-trihydroxyestra-1,3,5(10)7-tetraene-6,17-dione).

Once released into the environment, bile salts are subject to bacterial degradation [4,5,6] (Figure 1A). As shown for the model organisms *Pseudomonas stutzeri* Chol1 [7], *Comamonas testosteroni* TA41 [8], and *Rhodococcus jostii* RHA1 [9], bile salts can be degraded via the 9,10-*seco*-pathway for steroid degradation that involves Δ^1,4^-3-keto intermediates and will therefore be referred to as Δ^1,4^-variant (see the work of [4,6,10] and references therein): In the first steps of this well-elucidated pathway, the A-ring of the steroid skeleton is oxidized to a Δ^1,4^-3-keto-structure (III in Figure 1). Simultaneously, the side chain is degraded via β-oxidation and aldolytic reactions leading to side chain-less androsta-1,4-diene-3,17-diones (ADDs; e.g., 12β-DHADD, IV in Figure 1, in the degradation of cholate). 9α-hydroxylation of ADDs by the monooxygenase complex KshAB leads to cleavage of the B-ring upon A-ring aromatization and yields 9,10-*seco*-steroids such as 3,7,12-trihydroxy-9,10-*seco*-androsta-1,3,5(10)-triene-9,17-dione (THSATD, V) [11,12,13,14]. The A-ring is cleaved by consecutive hydroxylation and *meta*-cleavage similar to degradation of aromatic compounds [15,16,17,18]. The intermediate with opened A- and B-rings is then further degraded via hydrolytic and β-oxidation reactions (see [6,10] and references therein). Genes for degrading bile salts via this pathway are widespread in many bacterial species and metagenomes from different environments [19,20].

Recently, an alternative variant for the degradation of 7-hydroxy bile salts such as cholate (I in Figure 1) was found in *Dietzia* sp. strain Chol2 and *Sphingobium* sp. strain Chol11, formerly *Novosphingobium* sp. strain Chol11, that proceeds via intermediates with a 3-keto-Δ^4,6-^structure of the steroid skeleton and is therefore referred to as Δ^4,6^-variant [21,22,23]. After A-ring oxidation leading to Δ^4^-3-keto-cholate (II) during the degradation of cholate [24], a further double bond is inserted into the B-ring upon elimination of the 7OH by the dehydratase Hsh2 [22], leading to 12α-hydroxy-3-oxo-4,6-choldienoate (HOCDA, IX) as prominent intermediate. Side-chain degradation via a so far mostly unknown mechanism results in 12-hydroxy-androsta-1,4,6-triene-3,17-dione (HATD, X) [11,25]. Bioinformatic, proteomic, and first physiological analyses indicated that further degradation of HATD most probably proceeds via 9α-hydroxylation and 9,10-*seco*-cleavage [11]: In heterologous complementation experiments, expression of three of five homologs of the oxygenase subunit KshA of strain Chol11 in a *kshA* deletion mutant of *P. stutzeri* Chol11 lead to the production of the expected *seco*-steroid 3,12β-dihydroxy-9,10-*seco*-androsta-1,3,5(10),6-tetraene-9,17-dione (DHSATD, XI). However, this activity seemed to be very low. While the *seco*-steroid THSATD (V) is present as a dominant intermediate in *P. stutzeri* Chol1 culture supernatants [21], no DHSATD was reported for the supernatants of strain Chol11 cultures so far. This raises questions about the hypothesis of HATD 9α-hydroxylation as a central reaction during bile salt degradation in strain Chol11.

A characteristic of bacterial steroid degradation is the transient extracellular accumulation of intermediates that can be observed in laboratory cultures [21,26]. In soil slurry experiments, degradation of the dihydroxy bile salt chenodeoxycholate concomitant with the transient accumulation of at least Δ^1,4^-intermediates could be observed [27], indicating that extracellular accumulation of intermediates is a phenomenon also present in natural environments. Considering the high concentration of bile salts released with manure, the transient release of intermediates with endocrine effects may affect biota in agricultural soils [27,28]. In addition, this transient release may enable interspecies cross-feeding when different strains are present. Interspecies cross-feeding of, e.g., substrate compounds, vitamins, or H_2_ is a common principle in mixed bacterial communities [29,30,31] and therefore seems possible for steroid compounds. Cross-feeding experiments regarding bacterial bile salt degradation were so far only conducted by feeding purified intermediates to cultures of other strains [21]. *P. stutzeri* Chol1 is not able to completely degrade Δ^4,6^-intermediates such as HOCDA (IX) and only degrades the side chain, which results in HATD (X) [21]. HATD is then transformed to DHSATD (XI) as well as the dead-end product 1α,2α,12β-trihydroxy-androsta-4,6-triene-3,17-dione (THADD, XII) by two different types of hydroxylation reactions [21] that are both catalyzed by KshAB_Chol1_ [11]. It is unknown if cross-feeding of steroid metabolites would occur in bacterial co-cultures and if the dead-end products are degradable by other strains.

The goals of this study were first to further investigate 9α-hydroxylation in strain Chol11 by providing DHSATD as a substrate. As this resulted in the identification of a second side reaction leading to an unprecedented side product, the presence of both pathway variants in the soil, as well as effects of the known side product THADD and the newly discovered side product, were analyzed.

## 2. Material and Methods

### 2.1. Cultivation of Bacteria

Strains of *Pseudomonas stutzeri* Chol1 (DSM 103613) [7] and *Sphingobium* sp. strain Chol11 (DSM 110934) [21] were grown in the HEPES buffered mineral medium MB as described previously [21,29]. *P. stutzeri* Chol1, *Sphingobium* sp. strain Chol11 wt and Δ*nov2c349* were grown with 1 mM cholate as carbon source, *P. stutzeri* Chol1 pBBR1MCS-5::*hsh2* [22] and *P. stutzeri* Chol1 Δ*kstD1* Δ*stdA1* pBBR1MCS-5::*hsh2* [11] were grown with 12 mM succinate and *Sphingobium* sp. strain Chol11 Δ*sclA* [25] was grown with 15 mM glucose. *Escherichia coli* strains and most other strains containing pDM4 [32] or pBBR1MCS-5 [33] were cultivated in lysogeny broth medium (LB) [34] with respective antibiotics at 30 °C. For cultivating *E. coli* ST18 [35], 50 μg mL^−1^ 5-aminolevulinic acid were added. Strains containing pDM4 were cultivated with 30 or 90 μg mL^−1^ chloramphenicol, and strains containing pBBR1MCS-5::*hsh2* were cultivated with 20 μg mL^−1^ gentamicin. Strains were maintained on agar plates, prepared from the aforementioned media with 1.5% (*w*/*v*) Bacto agar (BD, Sparks, USA) and with either cholate for strains Chol1 and Chol11, glucose for mutant strains of strain Chol11, or succinate and gentamicin for strain Chol1 pBBR1MCS-5::*hsh2* and strain Chol1 Δ*kstD1* Δ*stdA1* pBBR1MCS-5::*hsh2.*

### 2.2. Growth Experiments and Co-Cultures

Growth experiments and co-cultures were performed in 3–5 mL medium in 10 mL test tubes at 30 °C and orbital shaking (Minitron or Ecotron, Infors HT, Einsbach, Germany). Precultures were grown with the respective carbon source for about 17 h and added to main cultures without previous washing. DHSATD (XI in Figure 1) was added in concentrations equaling the two-fold concentration produced in cultures of *P. stutzeri* Chol1 pBBR1MCS-5::*hsh2* cultivated with 1 mM cholate. MDTETD (XIII) was added in concentrations equaling the ten-fold concentration produced in co-cultures of *P. stutzeri* Chol1 pBBR1MCS-5::*hsh2* and *Sphingobium* sp. strain Chol11 Δ*sclA* cultivated with 1 mM cholate. Growth was tracked by measuring the optical density at 600 nm (OD_600_) (Camspec M107, Spectronic Camspec, UK). Samples for HPLC-MS measurements were withdrawn at defined time points.

### 2.3. Cell Suspension Experiments

For cell suspensions of *Sphingobium* sp. strain Chol11, a preculture with 1 mM cholate or 15 mM glucose was incubated for 6 h. Main cultures containing the same carbon source were seeded with the preculture to OD_600_ = 0.015 and incubated at 30 °C with orbital shaking at 200 rpm for about 16 h. In the exponential growth phase, cells were harvested by centrifugation at 8000× *g* and 4 °C for 8 min. Cells were washed and resuspended in MB medium without a carbon source. Cell suspensions were diluted to defined OD_600_ values. Samples for HPLC-MS measurements were withdrawn instantly after adding DHSATD (concentration as described) or cholate (1 mM) and at further time points thereafter.

For anoxic cell suspension experiments, anoxic 10 mL test tubes with butyl rubber plugs were prepared by flushing 10 min with sterile N_2_. Sterile syringes were used for apportioning cell suspensions, adding DHSATD, and taking samples.

For testing inhibiting conditions, 1 mL cell suspension with an OD_600_ of 0.155 was filled into a 2 mL plastic tube (Sarstedt, Nümbrecht, Germany). Pasteurization was carried out in these plastic tubes by incubation at 90 °C for 10 min. MB without carbon sources was used as sterile control. A total of 1 mM CuSO_4_ was added from a 100 mM stock solution. Water was added as CuSO_4_ control. The tubes were incubated for 4 to 5 days at 30 °C without shaking.

### 2.4. Abiotic Transformation of Steroid Compounds

DHSATD (XI in Figure 1) was incubated in sterile MB at different pH values and oxygen availabilities. Different pH values were adjusted with 1 M HCl or 32% NaOH. DHSATD was diluted in the respective MB to concentrations equaling the six-fold concentration produced in cultures of *P. stutzeri* Chol1 pBBR1MCS-5::*hsh2* cultured with 1 mM cholate, apportioned into 500 μL portions in 1.5 mL plastic tubes (Sarstedt, Nümbrecht, Germany) and incubated at 30 °C. HPLC samples were withdrawn directly after mixing and at defined time points thereafter.

The same DHSATD concentration in 1 mL MB at pH 7 was incubated in 10 mL HPLC glass vials (Thermo Fisher Scientific, Waltham, Massachusetts, USA) with butyl rubber plugs and crimp caps that were either only autoclaved or autoclaved and subsequently flushed with N_2_. Filling and taking samples were conducted with sterile syringes. The vials were incubated at 30 °C and 200 rpm.

### 2.5. Enrichment of Bacteria

Samples from soil and manure of different sites and animals, as well as water samples from a duck pond, were used for enrichment cultures. Samples were resuspended with Milli-Q pure water (Merck Millipore, Darmstadt, Germany) if necessary and diluted 10^3^ to 10^9^ in Milli-Q water. A total of 100 μL of each dilution were used to seed 5 mL of MB with MDTETD (XIII in Figure 1). Enrichment cultures were incubated at 30 °C with rotary shaking at 200 rpm for several weeks. A total of 100 μL of turbid cultures were transferred into fresh 5 mL MB with MDTETD. HPLC-MS samples were withdrawn on a regular basis.

### 2.6. Soil Microcosms

Soil microcosms were set up by mixing 1 g soil collected from various agriculturally used fields in the Münsterland region with 0.5 mL either 1 mM cholate or 1 mM HOCDA (VIII in Figure 1) dissolved in sterile Milli-Q pure water in a 2 mL plastic tube (Sarstedt, Nümbrecht, Germany). The microcosms were incubated at room temperature and inverted once a day. At several time points, HPLC-MS samples were withdrawn by centrifugation of plastic tubes at >16,000× *g* for 5 min at room temperature. For every sample, one tube was sacrificed. Supernatants were stored at −20 °C until extraction for HPLC-MS measurements.

### 2.7. Cloning Techniques and Construction of Unmarked Gene Deletions

The unmarked deletion mutant *Sphingobium* sp. strain Chol11 Δ*nov2c349* (NCBI accession number *WP_097093565*) was constructed as described [24] with the help of splicing by overlapping extension PCR (SOE-PCR) [36]. Up- and downstream DNA segments were amplified with the help of primer pairs upfor/uprev and dnfor/dnrev, respectively (Table 1). The fragments were assembled by SOE-PCR and amplified with the help of primer pair upfor/dnrev. The resulting fragment was ligated into the suicide vector pDM4, and strain ST18 colonies containing pDM4 with insert were identified by colony PCRs with primer pair pDM4_MCS_for/rev. The vector was transferred to strain Chol11 by conjugation, and strain Chol11 colonies with an inserted vector were identified with colony PCRs to test for pDM4 backbone. Unmarked gene deletion after sucrose selection was confirmed by PCR and subsequent sequencing with primer pair upfor/dnrev.

### 2.8. Preparation of Steroid Compounds

Cholate from ox or sheep bile (≥99%) was purchased from Sigma-Aldrich (St. Louis, MO, USA).

Steroid compounds were produced by biotransformation in 500 to 600 mL MB or the phosphate-buffered MMChol medium [7] in 2 L Erlenmeyer flasks at 30 °C and rotary shaking at 130 up to 200 rpm. For production of a DHSTAD/THADD (XI/XII in Figure 1) mixture or HOCDA (IX), *P. stutzeri* Chol1 pBBR1MCS-5::*hsh2* [22] or *P. stutzeri* Chol1 Δ*kstD1* Δ*stdA1* pBBR1MCS-5::*hsh2* [11], respectively, were incubated with cholate and the respective antibiotics until cholate was completely transformed. For the production of MDTETD (XIII), *P. stutzeri* Chol1 pBBR1MCS-5::*hsh2* was grown with 2 mM cholate without antibiotics until cholate was completely transformed into DHSATD and THADD. *Sphingobium* sp. strain Chol11 Δ*sclA* was added to the culture and incubated until DHSATD and THADD were completely transformed to MDTETD. Supernatants were harvested and extracted with ethyl acetate [7,37]. Supernatants containing HOCDA or MDTETD were acidified with HCl to gain a pH of about 2–3 before extraction. Steroid compounds were resolved in Milli-Q pure water (Merck, Darmstadt, Germany) and sterilized by filtration (0.2 μm cellulose acetate filter, VWR, Radnor, PA, USA). The concentration of HOCDA was determined as described [21]. The concentration of DHSATD and MDTETD was determined in relation to the cultures they were extracted from. If not indicated otherwise, DHSATD or MDTETD were used in further experiments in a two-fold or ten-fold concentration, respectively, compared to the original culture. For the production of pure DHSATD or THADD, they were purified from a DHSATD/THADD mixture by semi-preparative HPLC and extraction from the eluate. For NMR measurements, MDTETD was prepared as described and purified by two steps of semi-preparative HPLC. MDTETD was extracted from the eluate, resolved in methanol, and dried prior to NMR measurements.

### 2.9. HPLC-MS Analysis

Steroid compounds and culture supernatants were analyzed by HPLC-MS measurements with a Dionex Ultimate 3000 HPLC (Thermo Fisher Scientific, Waltham, Massachusetts, USA) with a UV/visible light diode array detector and a coupled ion trap mass spectrometer (Amazon speed, Bruker; Bremen, Germany) with an electro-spray ion source (ESI) as described previously [22] with only negative mode MS. All bile salts and their degradation intermediates were analyzed using a reversed phase C18 column (Knauer Wissenschaftliche Geräte, Berlin, Germany; 150 × 3 mm, Eurosphere II, 100-5 C18) at 25 °C. Ammonium-acetate buffer (10 mM, pH 6.7) and acetonitrile were used as eluents with a flow rate of 0.3 mL min^−1^ as described previously [22]. For purification of DHSATD, THADD and MDTETD, a reversed phase C18 column (Knauer Wissenschaftliche Geräte, Berlin, Germany; 250 × 8 mm, Eurosphere II, 100-5 C18) and ammonium-acetate buffer and acetonitrile were used. For separating DHSATD and THADD, a gradient method with a flow rate of 2.5 mL min^−1^ starting at 20% acetonitrile for 2 min, increasing to 60% acetonitrile within 15 min and returning to 20% acetonitrile within 1 min was used, after which the column was equilibrated for 5 min. For purification of MDTETD, a gradient method starting at 20% acetonitrile with a flow rate of 2 mL min^−1^ for 2 min, increasing to 60% acetonitrile within 13 min, maintaining 60% acetonitrile for 0.2 min with a flow rate of 3 mL min^−1^ and returning to 20% acetonitrile within 0.8 min and a flow rate of 3 mL min^−1^ was used, after which the column was equilibrated for 4 min.

The purity of synthesized compounds and concentrations of DHSATD and MDTETD for further investigations were assessed by HPLC-MS measurements of stock solutions.

Cholate concentration as well as the concentration of THSATD (V in Figure 1), DHSATD (XI), THADD (XII), and MDTETD (XIII) was determined as peak area from base peak chromatogram in negative mode MS measurements. HOCDA (IX) concentration was determined as peak area from extracted ion chromatogram with 385 Da in negative mode MS measurements. Intermediate identification, as well as structure assignments, were performed due to molecular masses and UV absorption spectra as well as retention time.

Most samples were gained as supernatants by centrifugation at >16,000× *g* and room temperature for 5 min. Supernatants were stored at −20 °C until measurement and centrifugated again prior to measurements. Samples from soil microcosms were purified by organic extraction prior to analysis by HPLC-MS measurements. For this, 200 μL of the sample were acidified with 30 μL 1 M HCl to gain pH 1–2 and extracted with 600 μL ethyl acetate. Ethyl acetate was dried off, and samples were resolved in 150 μL ethanol. All extracted samples were analyzed by measurements as described.

### 2.10. NMR Analysis of MDTETD

NMR spectra for structural elucidation of MDTETD were acquired on a Bruker Avance III spectrometer equipped with a 5 mm BBI H/X double resonance broadband probe with Z-gradient at a proton Larmor frequency of 600 MHz. A dry sample of MDTETD was dissolved in the deuterated methanol (Deutero GmbH, Kastellaun, Germany) to a concentration of 16 mg/mL and transferred to the 5 mm NMR tube. One-dimensional ^1^H and ^13^C spectra were acquired with a spectral width of 20 ppm in the ^1^H and 200 ppm in the ^13^C dimension with 65,536 data points. Two-dimensional ^1^H-^13^C-HSQC, HMBC, COSY, TOCSY, and NOESY experiments were recorded with 1024 increments, 4096 detected data points each. Spectral width in the ^13^C dimension in the ^1^H-^13^C-HSQC and HMBC spectra was set to 165 and 222 ppm, respectively. NOESY mixing time was set to 500 ms. 1,1-ADEQUATE and 1,n-ADEQUATE experiments were acquired with 256 increments of 2048 data points each and had a spectral width of 100 ppm in the ^13^C dimension.

One-dimensional heteronuclear nuclear overhauser effect (HetNOE) experiment was performed on the Bruker Avance III 600 MHz spectrometer equipped with a 5 mm TBO H/X double resonance broadband probe with Z-gradient. HetNOE spectra were recorded with 131,072 data points, and up to 32,768 scans were acquired.

For processing of the two-dimensional spectra, a squared sine bell window function, as well as zero filling to double the amount of the acquired data points, were used in both dimensions. Bruker TopSpin was used to acquire (v2.5), process, and analyze (v3.5) the spectra.

### 2.11. Modified Zebrafish Embryo Toxicity Test and Transcriptomics

A modified zebrafish embryo toxicity test (ZFET) (OECD236) was performed as described previously [38]. For THADD and MDTETD, nominal concentrations of 100 and 1000 µg/L were tested against non-treated controls in triplicates. Test concentrations were approximate maximum concentrations and based on the calculations in Figure S6 and weight of probably residual water-containing solid samples. Test solutions were prepared in copper-reduced tap water, and pH was adjusted to ~7.5. RNA sequencing and differential gene expression analysis was performed as described previously [38]. Raw and processed data have been deposited in the ArrayExpress database at EMBL-EBI (www.ebi.ac.uk/arrayexpress) (accessed on 11 October 2021) [39] under accession number E-MTAB-10922. The DEG analysis script is publicly available under: https://github.com/hreinwal/DESeq2Analysis (accessed on 11 October 2021). Overrepresentation analysis (ORA) was performed for gene ontology (GO) terms [40] in R using ClusterProfiler v3.18 [41] and ReactomePA v1.34 [42]. Gene clusters were analyzed with compareCluster() default settings and BH p-value correction.

## 3. Results

### 3.1. Ring Cleavage Intermediate DHSATD Transiently Accumulates in Supernatants of Sphingobium sp. Strain Chol11 in Very Low Concentrations

**Figure 2:**
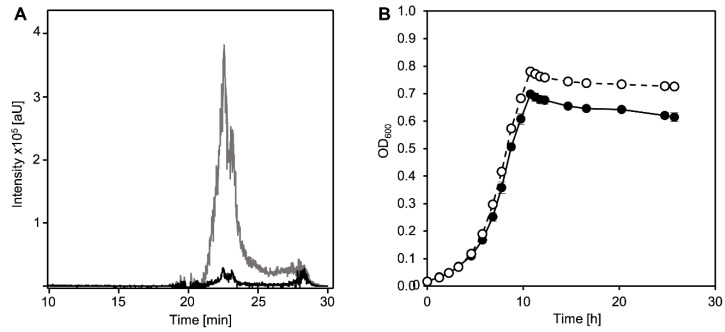
(**A**) Detection of DHSATD (XI) in supernatants of cultures of *Sphingobium* sp. strain Chol11 wt (black line) and the deletion mutant *Sphingobium* sp. strain Chol11 Δ*nov2c349* (gray line) during growth with cholate after 5.7 h of incubation. HPLC-MS data are displayed as extracted ion chromatogram at negative ion mode of MS (*m*/*z* value of DHSATD ([M-H]^−1^ = 313 Da)).(**B**) Growth of *Sphingobium* sp. strain Chol11 wt (filled circles) and *Sphingobium* sp. strain Chol11 Δ*nov2c349* (open circles) with 1 mM cholate (I in Figure 1) as sole carbon source. Error bars indicate standard deviation, which may not be visible if too small (*n* = 3).

Although all previous investigations hinted at DHSATD (XI in Figure 1) as an intermediate of cholate degradation in *Sphingobium* sp. strain Chol11 [11,23,25], this compound had never been detected in cultures of *Sphingobium* sp. strain Chol11. However, the evaluations of HPLC-MS measurements of culture supernatants were usually conducted with base peak chromatograms, in which small peaks may be concealed by other intermediates and background noise. Indeed, a specific search for the respective mass with the help of extracted ion chromatograms and specific absorbances revealed a transient accumulation of very low concentrations of DHSATD in culture supernatants of *Sphingobium* sp. strain Chol11 during growth with cholate (Figure 2A). Additionally, DHSATD could be detected in very low amounts when cell suspensions (OD_600_ = 0.4) of *Sphingobium* sp. strain Chol11 were supplemented with cholate (Figure S1).

To further support this, the unmarked deletion mutant *Sphingobium* sp. strain Chol11 Δ*nov2c349* was constructed. Nov2c349 (NCBI accession number WP_097093565) has 40% identity to the 9,10-*seco*-steroid (e.g., THSATD, V in Figure 1) monooxygenase component TesA2 from *C. testosteroni* [16] and is encoded in a large steroid degradation cluster of *Sphingobium* sp. strain Chol11, and nearly all enzymes encoded in this cluster are present in significantly higher (at least 1.5× increased) abundances during growth with bile salts compared to growth with control substrates [23]. This indicates that Nov2c349 might be the oxygenase component of a putative DHSATD processing enzyme. Interestingly, *Sphingobium* sp. strain Chol11 Δ*nov2c349* did not show any altered phenotype compared to the wild type regarding growth on cholate (Figure 2B). However, the strain transiently accumulated DHSATD in considerably higher amounts than the wild type (Figure 2A).

### 3.2. Cholate Degradation in Co-Cultures of Sphingobium sp. Strain Chol11 and P. stutzeri Chol1 Results in Accumulation of a Novel Steroid Compound

To further investigate the potential role of DHSATD (XI in Figure 1) in the cleavage of the steroid skeleton, we aimed to offer it as a substrate to *Sphingobium* sp. strain Chol11. The easiest way of producing DHSATD is expressing the 7α-hydroxysteroid dehydratase Hsh2 in *P. stutzeri* Chol1; this strain produces DHSATD and THADD (XII), as side products during growth with cholate [22]. To avoid tedious collecting of DHSATD and to rather provide it in a continuous way, we decided to use a co-culture approach, in which an Hsh2-producing strain of *P. stutzeri* Chol1 was co-incubated with strain *Sphingobium* sp. strain Chol11. For enforcing *Sphingobium* sp. strain Chol11 to use DHSATD as a potential substrate instead of cholate, *Sphingobium* sp. strain Chol11 Δ*sclA* was chosen*,* which can only slowly degrade steroids with a C_5_ side chain but is not affected in growth with steroids without side chain [25] (Figure 1B).

**Figure 3:**
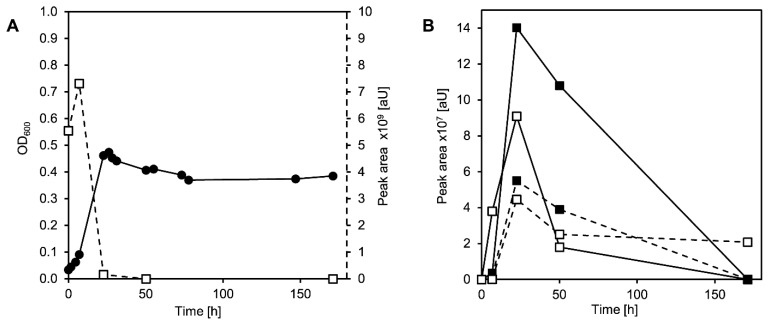
(**A**) Growth (filled circles) of a co-culture of *Pseudomonas stutzeri* Chol1 pBBR1MCS-5::*hsh2* and *Sphingobium* sp. Chol11 Δ*scl1* with cholate (open squares, second axis). (**B**) Formation of THADD (XII in Figure 1; filled squares, continuous line), DHSATD (XI; open squares, continuous line), THSATD (V; filled squares, dotted line), and MDTETD (XIII; open squares, dotted line). This figure shows the results of a single experiment representative for at least three reproducible experiments.

**Figure 4:**
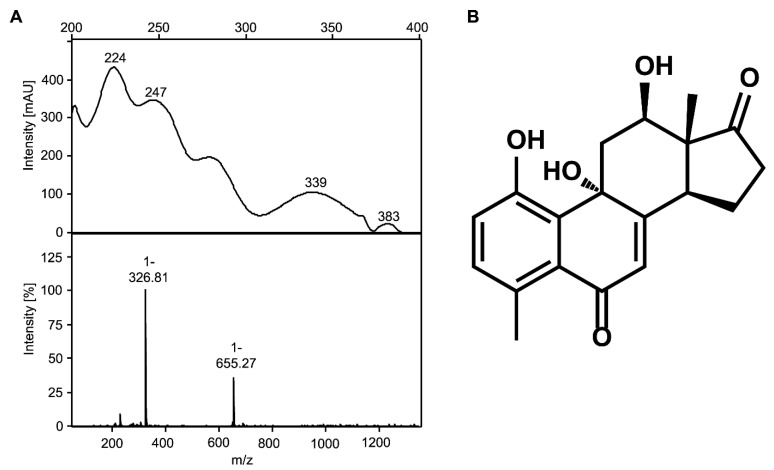
(**A**) UV (top) and mass (bottom) spectra of purified MDTETD. (**B**) Structure of MDTETD according to NMR spectroscopy; chemical shifts are listed in Table S1.

In this co-culture, cholate completely disappeared from the supernatant within 24 h, and the co-culture grew to an OD_600_ of nearly 0.5 in the same time span (Figure 3A). This value is lower than the OD_600_ reached by both wild-type strains with the same cholate concentration [7,21], indicating less efficient use of the carbon source. In the co-cultures, THSATD (V) as a known intermediate from Δ^1,4^-degradation as well as the expected dead-end products of *P. stutzeri* Chol1 pBBR1MCS-5:*hsh2,* DHSATD (XI) and THADD (XII), transiently accumulated in the culture supernatant (Figure 3B). All three steroid compounds were present at the highest concentration after about 24 h of incubation and were completely degraded after more than 150 h. Interestingly, an unknown metabolite accumulated as a dead-end product in this co-culture, which has never been observed in either single culture. The new metabolite appeared in HPLC-MS measurements with *m*/*z* 327 for [M-H]^−^ indicating a molecular mass of 328 Da and a UV spectrum clearly different from so far known steroid intermediates (Figure 4A).

### 3.3. The Novel Steroid Compound Named MDTETD Has an Unusual Ring Structure

By NMR analysis of the unknown compound, the structure of the steroid rings D, and partly C could be found using a combination of ^1^H-^13^C-HSQC and HMBC experiments. Typical ^13^C chemical shifts of methyl group C-18, carbonyl group C-17, and hydroxyl bound C-12 confirmed the presence of a structural motive of DHSATD (XI in Figure 1) in the structure. UV spectrum and ^1^H resonances in the aromatic region indicated the presence of several conjugated double bonds in the compound. Using HSQC, HMBC, NOESY, and COSY spectra, a number of isolated spin systems were generated, but because of the large number of quaternary carbons in the structure, there was not enough reliable connectivity information to combine these spin systems together. Additional strong insights into the carbon bond connections were delivered through the 1,1-ADEQUATE and 1,n-ADEQUATE experiments [43]. It was obvious from the new data that the methyl group C-19 could not be at the typical position between rings A and B. Considering long-range ^1^H-^13^C correlations of the methyl group C-19 and aromatic protons, it was proposed that the ring A was 180° rotated along the bond between C-5 and C-6 to form the structure indicated in Figure 4B. All resonances in the structure could be assigned. The structure was validated through the correlations in the NOESY spectrum and using heteronuclear NOE effects between methyl group C-19 and carbon atoms C-4 and C-5. Finally, the configuration of the chiral centers C-9, C-12, C-13, and C-14 was identified through the NOE connections in the structure. According to these results, the new metabolite was identified as 4-methyl-3-deoxy-1,9,12-trihydroxyestra-1,3,5(10)7-tetraene-6,17-dione (MDTETD, XIII in Figure 1), which has a complete steroid skeleton with a 180° rotated aromatic A-ring (Figure 4B, Table S1).

### 3.4. MDTETD Is Formed by Sphingobium sp. Strain Chol11 When Incubated with DHSATD

**Figure 5:**
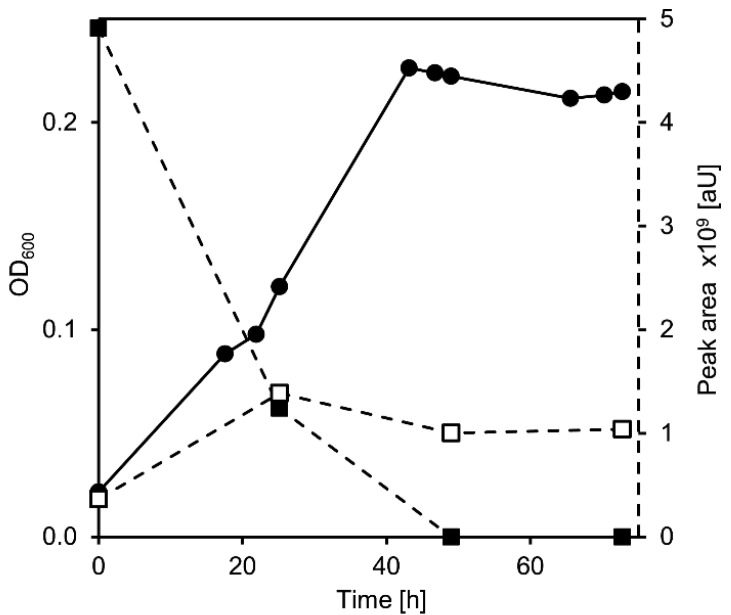
Growth of *Sphingobium* sp. strain Chol11 with DHSATD (XI in Figure 1, filled circles), degradation of DHSATD (filled squares, second axis), and accumulation of MDTETD (XIII, open squares, second axis) during growth with DHSATD. Error bars indicate standard deviation, which may not be visible if too small (*n* = 3).

In the next step, it was investigated whether one of the steroids appearing in the supernatants of the engineered co-culture was the precursor of MDTETD (XIII in Figure 1). As MDTETD formation has never been observed in mono-cultures of *P. stutzeri* Chol1 or its derivative with pBBRMCS-5::*hsh2*, these experiments were restricted to *Sphingobium* sp. strain Chol11. Additionally, THSATD (V) had been shown to be a natural intermediate during cholate degradation in *Sphingobium* sp. strain Chol11 Δ*hsh2* and therefore was not considered as a precursor [22]. Thus, experiments with purified DHSATD (XI) and THADD (XII) as substrates were performed. Notably, highly concentrated DHSATD preparations turned out to always be slightly contaminated with some MDTETD that could not be removed or appeared again after removal. *Sphingobium* sp. strain Chol11 grew to a final OD_600_ of more than 0.2 with DHSATD in a two-fold concentration compared to that in the engineered co-culture (Figure 5). DHSATD was completely degraded within about 50 h, accompanied by the accumulation of MDTETD (Figure 5).

In contrast, *Sphingobium* sp. strain Chol11 did apparently not grow with THADD; this compound was partly removed from the supernatant, but MDTETD was not formed (Figure S2). These results strongly indicated that DHSATD is the precursor of MDTETD.

### 3.5. MDTETD Is Produced from DHSATD by Sphingobium sp. Strain Chol11 via so far Unknown Reactions

**Figure 6:**
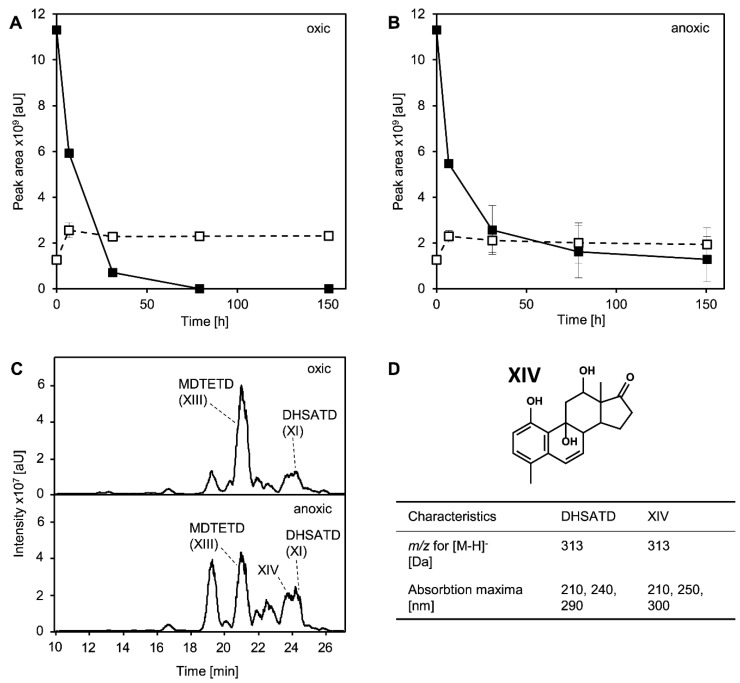
Degradation of DHSATD (XI in Figure 1, closed squares) and formation of MDTETD (XIII, open squares) by suspensions of cholate-grown cells of *Sphingobium* sp. strain Chol11 (initial OD_600_ = 0.13) under oxic (**A**) and anoxic (**B**) conditions. (**C**) HPLC-MS analyses of supernatants after 31 h of oxic (top) and anoxic (bottom) incubation. MS base peak chromatograms measured in negative mode are shown. (**D**) Proposed structure for a steroid compound named XIV found in cell suspensions incubated anoxically with DHSATD and comparison of characteristics of DHSATD and XIV. The structure suggestion is based on *m*/*z* values and absorption spectra. Error bars indicate standard deviation, which may not be visible if too small (*n* = 3).

In biotransformation experiments with dense cell suspensions instead of the low cell densities found in growth experiments, cholate-grown cells of *Sphingobium* sp. strain Chol11 degraded DHSATD (XI in Figure 1) in about 24 h while a small amount of additional MDTETD was formed (Figure 6A). Experiments with glucose-grown cells showed very similar results (data not shown). As DHSATD has to be hydroxylated during transformation to MDTETD, cell suspensions were also incubated anoxically to investigate the impact of O_2_. Under these conditions, DHSATD was only incompletely degraded within 150 h, while almost no additional MDTETD was formed (Figure 6B). Interestingly, these conditions led to an accumulation of various steroid compounds that might be intermediates between DHSATD and MDTETD (Figure 6C,D) according to their UV- and mass spectra. For one of these intermediates, a structure could be proposed based on its UV spectrum and its *m*/*z* value, which would agree with a direct precursor of MDTETD without hydroxyl group at C-6 (Figure 6D, XIV). Pasteurized cells showed no degradation of DHSATD and no formation of MDTETD (not shown). Cells inhibited with CuSO_4_ showed decreased DHSATD degradation and strongly increased MDTETD formation, while sterile controls containing CuSO_4_ were similar to sterile controls without CuSO_4_ (Figure S3).

To test if DHSATD (XI) can abiotically be transformed to MDTETD (XIII), DHSATD was incubated sterilely under different conditions. In contrast to cultures of *Sphingobium* sp. strain Chol11, no additional MDTETD was formed within 80 h when DHSATD was incubated in sterile medium at pH 7 or 8 (Figure S4A,B). In contrast, incubation at pH 9 led to strongly increased MDTETD concentrations (concentration doubled within 80 h) (Figure S4C). While DHSATD seemed to be stable at pH 7, DHSATD vanished from the supernatant of medium with pH 8 and 9, and a purple-colored precipitate formed. No difference could be observed for oxic and anoxic incubation of DHSATD at pH 7 (Figure S4D).

### 3.6. MDTETD Is Not Degraded in Enrichment Cultures and May Affect Physiological Functions of Fish

Neither *P. stutzeri* Chol1 nor *Sphingobium* sp. strain Chol11 were able to degrade MDTETD (XIII in Figure 1) (not shown). For investigating whether MDTETD is generally biodegradable, enrichment cultures were set up with inocula from various sites where bile salt degradation and cross-feeding may take place, such as manure, manured fields, and water from a duck pond. No MDTETD degradation was observed in any enrichment cultures, not even in those that became turbid after several weeks (Figure S5).

**Figure 7:**
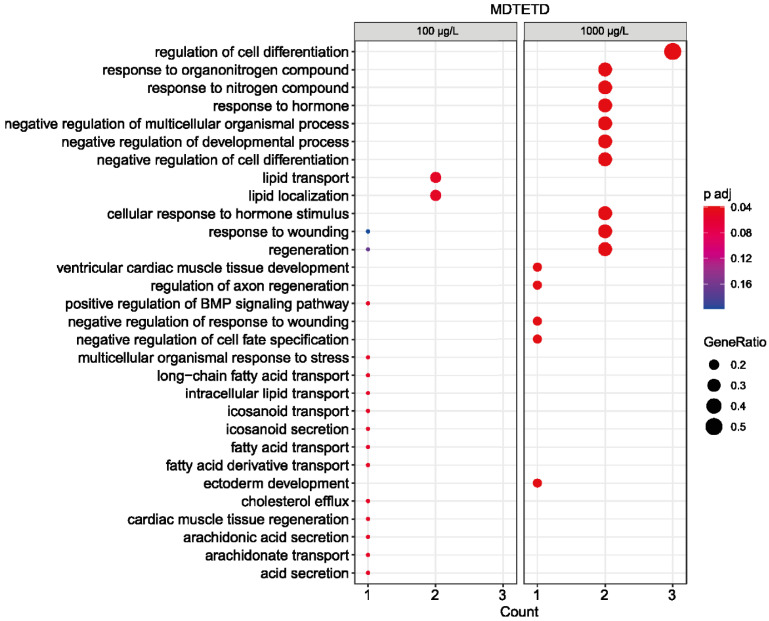
Overrepresentation analysis of GO biological processes based on statistically differentially expressed genes (padj ≤ 0.05) in zebrafish embryos after exposure to nominal concentrations of 100 µg/L (left panel) and 1000 µg/L MDTETD (right panel). The adjusted *p*-value is indicated as a color code, and the ratio of differentially expressed genes in each condition to the total number of genes assigned to each biological process is indicated by the dot size. The number of statistically differentially expressed genes assigned to each biological process for each condition is shown below.

The structure of MDTETD resembles ecdysteroid insect hormones [44,45,46] as well as vertebrate estrogen and aromatase inhibitors [47,48]. In addition, THADD (XII), the other side product formed in the co-culture, has some similarities to different androgenic compounds or aromatase inhibitors [47,48,49]. For investigating whether these compounds could induce endocrine effects in vertebrates, they were submitted to a modified zebrafish embryo toxicity test followed by transcriptome profiling. This approach has proven successful for detecting gene expression changes preceding endocrine modes of action previously [38]. For both compounds, which could be supplied in low amounts due to their very restricted availability, no macroscopic effects and only very moderate effects in the expression of target genes for aromatase inhibitors were observed. However, the transcriptome analysis of the higher dose of MDTETD revealed a low number of significant changes in the expression of a few genes (E-MTAB-10922, ArrayExpress database). Functional pathway enrichment showed that these genes were related to the regulation of cellular and multicellular differentiation development as well as to the response to (organo)nitrogen compounds and hormones (Figure 7).

### 3.7. Presence of Both Bile Salt Degradation Variants in Soils Indicates Potential for Cross-Feeding

**Figure 8:**
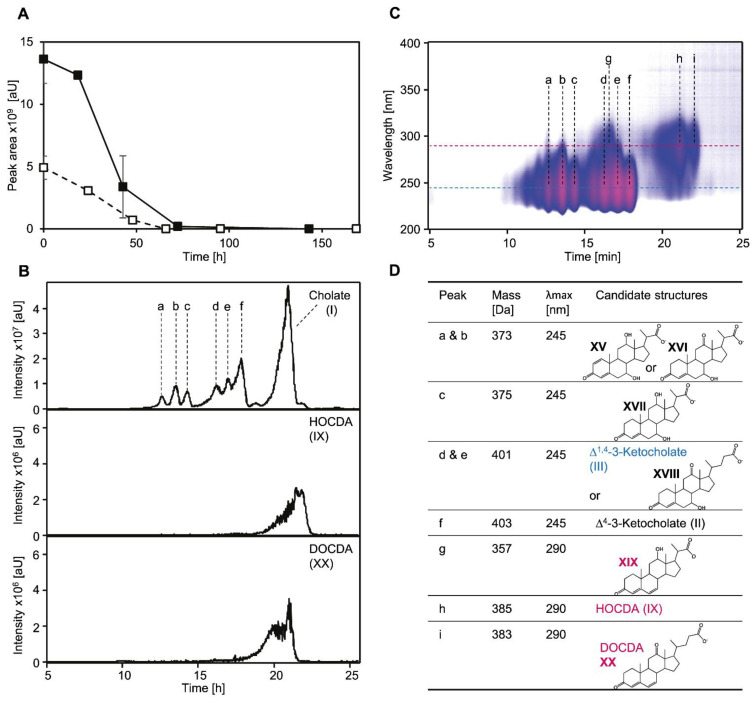
(**A**) Degradation of 1 mM cholate (solid line, filled squares) and 1 mM HOCDA (IX in Figure 1, dotted line, open squares) in soil slurry microcosms with soil samples from agriculturally used fields in the Münsterland region. Error bars indicate standard deviation (*n* = 3). (**B**) MS base peak chromatogram of the extracted supernatant of a soil slurry incubated with 1 mM cholate for about 48 h (top) as well as extracted ion chromatograms with the *m*/*z* values of HOCDA (383 Da for [M-H]^−1^, middle) and DOCDA (XX, 385 Da for [M-H]^−1^, bottom). Samples were measured in negative MS mode. (**C**) 3D UV chromatogram of the extracted supernatant of a soil slurry incubated with 1 mM cholate for about 48 h and structure suggestions for several intermediates assigned to peaks. Intensity is shown as a heat map. Red indicates highest absorption. (**D**) Candidate structures for peaks a-i found in (**B**,**C**). Masses and absorption maxima (λ_max_) were determined by HPLC-MS measurements. Structure suggestions are based on molecular masses, absorption spectra, and retention time. Candidate structures belonging (blue) to the Δ^1,4^-pathway, (red) to the Δ^4,6^-pathway, and (black) potentially occurring in both pathways. When structures could not be assigned unambiguously, two possible structures are depicted. XV: 7,12-Dihydroxy-3-oxo-pregna-4-ene-carboxylate, XVI: 7-Hydroxy-3,12-dioxo-pregna-4-ene-carboxylate, XVII: 7,12-Dihydroxy-3-oxo-pregna-4-ene-carboxylate, XVIII: ∆^4^-3,12-Diketocholate, XIX: DOCDA (12-Hydroxy-3-oxo-pregna-4,6-diene-carboxylate, XX: 3,12-Dioxo-4,6-choldienoate).

The prerequisite for the formation of MDTETD in, e.g., agricultural soils would be that bacteria using the Δ^4,6^-variant are present in such soils and show the transient release also in situ as observed for several bile salt-degrading-type strains of the *Sphingomonadaceae* under laboratory conditions [23]. For investigating bile salt transformation in soil, watery soil microcosms from soil samples taken in the Münsterland region in Germany were tested for cholate degradation and intermediate accumulation. Cholate disappeared from the supernatants within about four days without an apparent lag phase (Figure 8A). Additionally, several compounds were detected in the supernatants that could be identified as intermediates from both cholate-degradation variants by their characteristic absorption at 245 nm for Δ^1/4/1,4^-3-keto structures, and 290 nm for Δ^4,6^-3-keto structures, respectively, and structures could be assigned or suggested according to these absorption spectra as well as mass spectra and retention time (Figure 8B–D). In particular, 12β-DHADD (V in Figure 1) as well HOCDA (IX) as characteristic intermediates of Δ^1,4^ or Δ^4,6^ degradation, respectively, were found in the microcosms.

To further confirm the presence of bacteria degrading cholate via the Δ^4,6^-variant, the degradation of HOCDA in soil microcosms was monitored, which also disappeared from the supernatants within about four days (Figure 8A).

## 4. Discussion

Aerobic bacterial degradation of 7-hydroxy bile salts in soil and water can proceed via two pathway variants, namely the Δ^1,4^-variant and the Δ^4,6^-variant [6]. The Δ^4,6^-variant is prevalent in the *Sphingomonadaceae* and differs from the Δ^1,4^-variant, which is found in other Proteobacteria and Actinobacteria, especially in the degradation of the side chain [11,23], while the cleavage of the steroid skeleton was proposed to proceed via 9,10-*seco* cleavage in both variants. In *Sphingobium* sp. strain Chol11, DHSATD (XI) is the expected 9,10-*seco* intermediate, which can be formed from the C_19_-steroid HATD (X) by three Rieske-monoxygenases of *Sphingobium* sp. strain Chol11 expressed in a heterologous host [11]. In this study, we detected a transient accumulation of DHSATD supporting this hypothesis. Furthermore, increased DHSATD concentrations could be found in the cultures of *Sphingobium* sp. strain Chol11 Δ*nov2c349* lacking the DHSATD processing enzyme. However, there are also observations that contradict this conclusion. First, DHSATD concentrations were very low, and also the activities of the DHSATD-forming Rieske monooxygenase of *Sphingobium* sp. strain Chol11 were low compared to that from *P. stutzeri* Chol1 [11]. Furthermore, *Sphingobium* sp. strain Chol11 Δ*nov2c349* grew with cholate similarly to the wild type, although this enzyme is the only homolog for this reaction in the genome. Finally, when DHSATD was offered to *Sphingobium* sp. strain Chol11 as substrate, this led to the formation of the dead-end product MDTETD (XIII in Figure 1).

Our results strongly suggest that MDTETD is the product of side reactions catalyzed by *Sphingobium* sp. strain Chol11 when DHSATD (XI) is provided as a substrate in far higher concentrations than found during growth with cholate. As a molar extinction coefficient for DHSATD was not available, we were not able to exactly determine the concentration of DHSATD. Nevertheless, calculations using approximate molar growth yields of *P. stutzeri* Chol1 under different conditions indicate that about 0.2 to 0.6 mM DHSATD might be present in test cultures with *Sphingobium* sp. Chol11 and sterile controls (Figure S6).

The actual reactions leading to MDTETD remain unknown, and especially the closing of the B-ring of steroids via enzymatic mechanisms as a prerequisite for this conversion has not been described yet [50]. Unfortunately, the characterization of the reactions leading to the formation of MDTETD from DHSATD was impaired by the fact that DHSATD stock solutions always contained MDTETD. As DHSATD was purified by preparative HPLC and MDTETD could be easily eliminated by this, we suppose that DHSATD undergoes a slow chemical conversion to MDTETD when the substrate concentration is very high as in the purified stock solution (up to 6 mM according to calculations in Figure S6) because this chemical conversion is not observed at lower concentrations. A potential mechanism for this chemical conversion proceeds via rotation of the A-ring along the bond C-5–C-6, the closing of the B-ring by a Friedel-Crafts-reaction, and hydroxylation of C-6 (Figure S7). While DHSATD was stable at neutral pH, its concentration decreased at pH 9, accompanied by MDTETD formation and precipitation of a purple pigment. The latter suggests autoxidation of DHSATD, which could result in a polyphenol as reported before for similar steroid intermediates [7,18] forming the precipitate, but could also lead to the abiotic hydroxylation at C-6. From the aerobic degradation of estrogens by *Sphingomonas* sp. strain KC8, an abiotic side reaction of a *meta*-cleavage product with an opened A-ring with ammonium led to the formation of a pyridine derivative [51], which further indicates the possibility of abiotic side reactions with *seco*-steroids.

In the presence of *Sphingobium* sp. strain Chol11 cells, DHSATD degradation was possible at neutral pH, occurred at a higher rate than abiotically at pH 9, and caused much less pigment formation. Instead, a slight increase in MDTETD formation was detected, indicating that indeed cells of *Sphingobium* sp. strain Chol11 catalyzed this reaction. This is further supported by the fact that MDTETD was formed neither in cultures of *P. stutzeri* Chol1 under conditions that lead to the accumulation of DHSATD nor in sterile or pasteurized controls.

The fact that biotic MDTETD formation was decreased under oxygen-limited conditions suggests that a monooxygenase might be responsible for the biotic C-6-hydroxylation and, thus, is the main factor for the higher rate of biotic MDTETD formation. In agreement with this conclusion, the oxygen-limited conversions showed transient accumulation of metabolites, the spectrometric properties of which would fit the intermediates of the postulated conversion of DHSATD to MDTETD but still lack the additional hydroxyl group. Besides accidental side reactions, the production of MDTETD could be due to detoxification reactions as DHSATD may be toxic by itself, similar to THSATD [7]. In this respect, the C-6-hydroxylation might be catalyzed by a rather unspecific detoxifying cytochrome P_450_ monooxygenase as often found in the liver [52,53].

Apparently, *Sphingobium* sp. strain Chol11 is able to convert DHSATD in a productive way for using bile salts as growth substrates and in a non-productive way leading to MDTETD as a dead-end metabolite. Therefore, the very low DHSATD concentration (based on the calculations in Figure S6 more than 1000fold lower than in the test cultures for DHSATD transformation) found in culture supernatants might be the outcome of a regulatory mechanism to prevent the formation of the side product MDTETD. It might be possible that the function of DHSATD-degrading monooxygenase Nov2c349 is taken over by another oxygenase as cleavage of the A-ring resembles *meta*-cleavage of aromatic compounds [54], and *Sphingomonadaceae* are well-known for their impressive catabolic repertoire regarding aromatic and xenobiotic compounds [55,56]

As MDTETD was recalcitrant to biodegradation and also exhibited slight physiological effects in a fish embryo assay, its formation in soils and water might be of concern. In the laboratory, MDTETD formation was discovered as a product of cross-feeding between bacteria using the Δ^1,4^-variant and the Δ^4,6^-variant. This raises the question of whether this cross-feeding is a realistic scenario in natural habitats. Soil microcosm experiments showed that both pathway variants are present in soil and that the excretion of Δ^1,4^- and Δ^4,6^-intermediates is not a laboratory artifact but can also be found for soil microorganisms as already shown for the degradation of chenodeoxycholate via the Δ^1,4^-variant [27]. However, the production of MDTETD was observed in a co-culture of engineered strains, in which the metabolic pathways were disturbed toward the overproduction of DSHATD. As we did not detect any MDTETD in our soil microcosm experiments upon organic extraction of pore water (not shown), this might indicate that the conditions allowed efficient degradation of bile salts. Nevertheless, deterioration of microbial metabolism, including bile salt degradation, might be caused in agricultural soils by pesticides [57] and antibiotics originating from manure [58,59,60]. In this respect, CuSO_4_, which is used as a pesticide [61,62,63], may inhibit DHSATD degradation and may lead to the formation of MDTETD by impeding the normal route for DHSATD degradation via A-ring oxygenation [15,16,64]. This could also be the reason for the enhanced MDTETD formation in cells treated with CuSO_4_ and seems plausible as copper ions are known to inhibit oxygenases [65,66].

The formation of MDTETD is the second side reaction described for the degradation of bile salts besides the formation of THADD [11,21]. The possibility of such side reactions is an important new aspect in bacterial steroid degradation regarding its ecological issues as well as its biotechnological potential.

## Figures and Tables

**Table 1:** Primers used for cloning and construction of unmarked gene deletions. Underlined: restriction sites.

Name	Sequence	Restriction Sites
upfor_*nov2c349*	TTTTTTTTCTAGACGGTCTGCGAGAAGGTGAGG	*Xba*I
uprev_*nov2c349*	TCGCGCATATGGCATCTGGCA	
dnfor_*nov2c349*	TGCCAGATGCCATATGCGCGACATCTTCTCCATTGTAGGCGA	
dnrev_*nov2c349*	TTTTTTTGTCGACCGTAGAAGAGCTCCATCGGG	*Sal*I
pDM4_MCS_for	AAGATGTGGCGTGTTACGGT	
pDM4_MCS_rev	AGGCTCTGGGAGGCAGAATA	

## Data Availability

Transcriptomic data sets are available at the ArrayExpress database under the accession number E-MTAB-10922 (www.ebi.ac.uk/arrayexpress) (accessed on 11 October 2021). The DEG analysis script is available under: https://github.com/hreinwal/DESeq2Analysis, (accessed on 11 October 2021). All other data are included in the manuscript and supplemental material.

## Supplementary Materials

The following are available online at https://www.mdpi.com/article/10.3390/microorganisms9102146/s1, Table S1 NMR analysis of MDTETD in deuterated methanol. Figure S1 Transient accumulation of DHSATD in cell suspensions of *Sphingobium* sp. strain Chol11 fed with cholate. Figure S2 Growth *of Sphingobium* sp. strain Chol11 with THADD and degradation of THADD. Figure S3 Influence of CuSO_4_ on the degradation of DHSATD and on the formation of MDTETD by cell suspensions of *Sphingobium* sp. strain Chol11 during 5 d of incubation. Figure S4 Transformation of DHSATD and accumulation of MDTETD in sterile MB. Figure S5 HPLC-MS analyses of supernatants from enrichment cultures incubated with MDTETD as energy and carbon source. Figure S6 Calculation of the approximate DHSATD concentration in test cultures for MDTETD production. Figure S7 Proposed (bio)chemical reaction pathway from DHSATD to MDTETD via the potential intermediate XIV.
