# Supplementary material for: Investigations on the Degradation of the Bile Salt Cholate via the 9,10-Seco-Pathway Reveals the Formation of a Novel Recalcitrant Steroid Compound by a Side Reaction in Sphingobium sp. Strain Chol11
Source: Microorganisms. 2021 Oct 14;9(10):2146. doi: 10.3390/microorganisms9102146 (PMC8540908; doi:10.3390/microorganisms9102146)
Supplement: Supplementary file 1 [file microorganisms-09-02146-s001.zip › microorganisms-1401088-supplementary.pdf]

Table S1 NMR analysis of MDTETD (Figure 4B) in deuterated methanol. ^{1}H and ^{13}C chemical shifts of MDTETD.

Atom No.	δ(^{13}C), ppm	δ(^{1}H), ppm
1	153.9	-
2	120.6	7.04, d, 8.3 Hz
3	132.6	7.17, d, 8.3 Hz
4	132.1	-
5	127.7	-
6	186.3	-
7	123.8	6.15, d, 1.4 Hz
8	158.1	-
9	71.1	-
10	132.6	-
11	41.9	1.47, dd, 14.1 / 11.2 Hz
		3.13, dd, 14.1 / 4.5 Hz
12	67.1	4.38, dd, 11.2 / 4.5 Hz
13	54.2	-
14	42.9	3.44, ddd, 12.7 / 5.5 / 1.3 Hz
15	18.2	2.00, m
		2.19, m
16	34.9	2.37, dt, 18.7 / 9.3 Hz
		2.61, m*
17	218.2	-
18	6.1	0.84, s
19	22.0	2.64, s

s: singlet, d: doublet, t: triplet, m: multiplet, -: no proton at given position

*: multiplicity could not be identified because of the overlap with the signal from H19

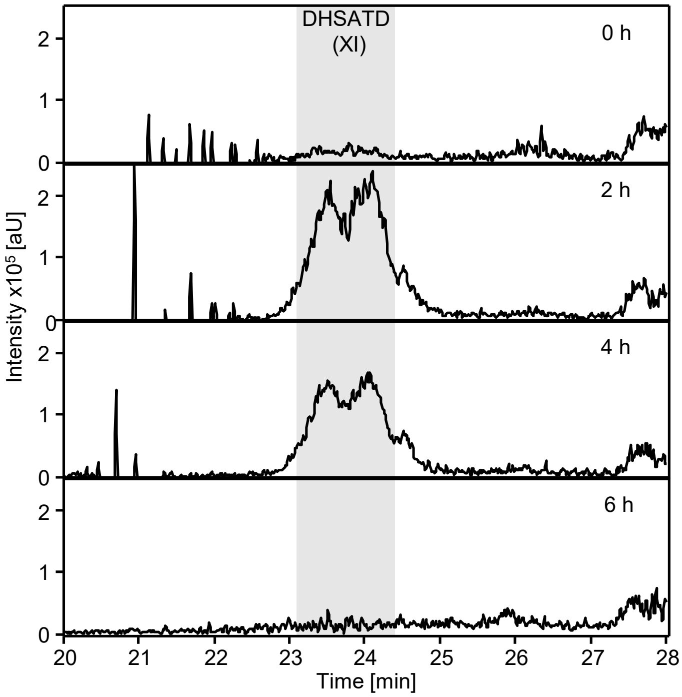

Figure S1 Transient accumulation of DHSATD (XI in Figure 1) in cell suspensions of *Sphingobium* sp. strain Chol11 (initial OD_{600} = 0.4) fed with 1 mM cholate. HPLC-MS data are displayed as extracted ion chromatogram at negative ion mode of MS (*m/z* value of DHSATD ($[M-H]^{-1}$ = 313 Da)). Grey: Expected retention time of the DHSATD peak.

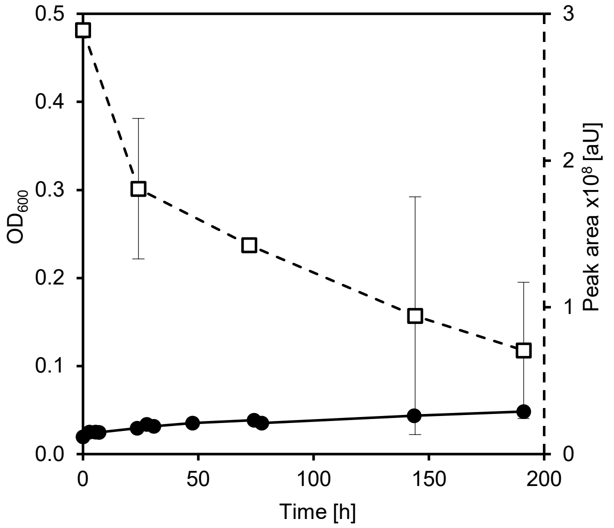

Figure S2 Growth *of Sphingobium* sp. strain Chol11 with THADD (XII in Figure 1) filled circles) and degradation of THADD (open squares, second axis). Error bars indicate standard deviation, which may not be visible if too small (n = 3).

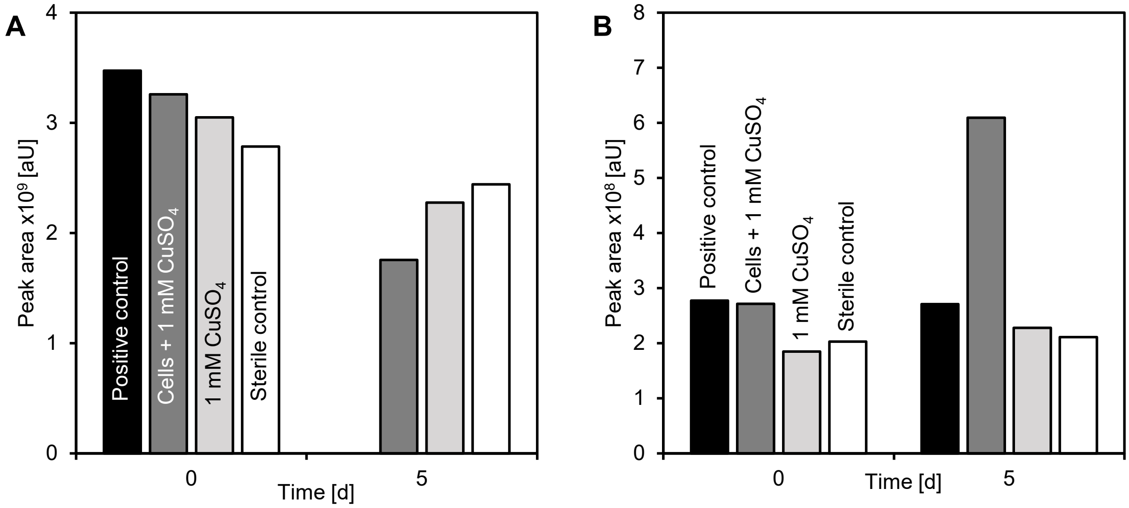

Figure S3 Influence of $CuSO_4$ on **(A)** degradation of DHSATD (XI in Figure 1) and on **(B)** formation of MDTETD (XIII) by cell suspensions of *Sphingobium* sp. strain Chol11 (OD_{600} = 0.15) during 5 d of incubation. Black: cell suspension without $CuSO_4$, positive control, dark grey: cell suspension treated with 1 mM CuSO4, light grey: sterile control with 1 mM $CuSO_4$, white: sterile negative control without $CuSO_4$. Bars indicate mean values of duplicates.

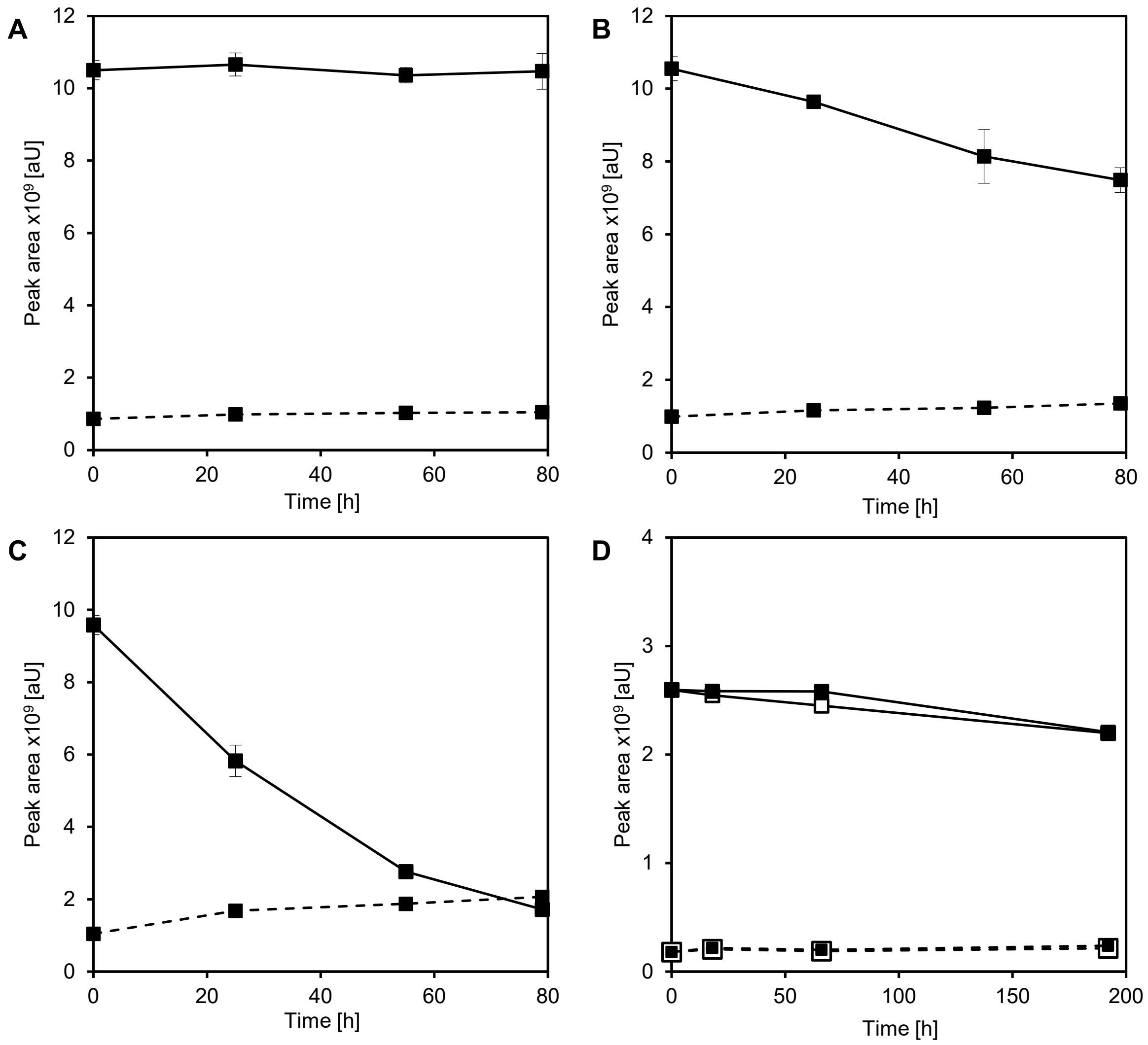

Figure S4 Transformation of DHSATD (XI in Figure 1; solid line) and accumulation of MDTETD (XIII; dotted line) in sterile MB. **(A-C)** Incubation in MB with pH values of 7 **(A)**, 8 **(B)** and 9 **(C)**. **(D)** Incubation under aerobic (closed squares) and anaerobic (open squares) conditions. Error bars indicate standard deviation, which may not be visible if too small (n = 3).

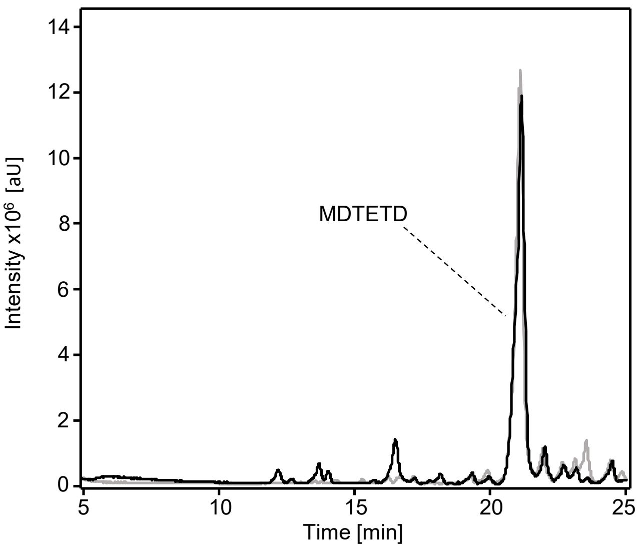

Figure S5 HPLC-MS analyses of supernatants from enrichment cultures incubated with MDTETD (XIII in Figure 2) as energy and carbon source. The inoculation material was obtained from an agriculturally used area. Black: 0 d, grey: 6 weeks. HPLC-MS data are displayed as base peak chromatograms in negative mode MS.

Cholate usage of *Pseudomonas stutzeri* Chol1:

Final OD_{600} of *P. stutzeri* Chol1 with 1 mM cholate

- under oxic conditions (complete degradation): 0.6 (1)
- under anoxic conditions (side chain degradation only): 0.15 (1)
- with pBBR1MCS-5::*hsh2* (partly complete degradation, partly transformation to dead end products): 0.45 (2)

Dead end products of *P. stutzeri* Chol1 pBBR1MCS-5::*hsh2* are
3,12β-dihydroxy-9,10-*seco*-androsta-1,3,5(10),6-tetraene-9,17-dione (DHSATD)
and 1α,2α,12β-trihydroxy-androsta-4,6-triene-3,17-dione (THADD),
which both have no side chain and a nearly complete steroid nucleus.
Therefore, it may be assumed, that *P. stutzeri* Chol1 pBBR1MCS-5::*hsh2* is only able to use the side chain of the cholate molecules transformed to DHSATD and THADD.

Calculation of fluxes to complete degradation and dead-end product formation in *P. stutzeri* Chol1:

Final OD_{600} of *P. stutzeri* Chol1 pBBR1MCS-5*::hsh2* =
(Amount of completely degraded cholate) * 0.6 +
(amount of cholate transformed to DHSATD and THADD = amount of cholate of which only the side chain is used) * 0.15

and

Total cholate concentration in cultures of *P. stutzeri* Chol1 pBBR1MCS-5::*hsh2* = 1 mM

⇒ (1) 0.45 = 0.6 * a + 0.15 * b
(2) a + b = 1

⇔ (1) 3 = 4 * a + b
(2) b = 1 – a

⇔ (1) 3 = 4 * a + 1 – a

⇔ (1) 2 = 3 * a

⇔ (1) a ≈ 0,67 mM (completely degraded)
(2) b ≈ 0,33 mM (transformed into DHSATD and THADD)

Calculation of DHSATD concentration in test cultures

In cultures of *P. stutzeri* Chol1 pBBR1MCS-5::*hsh2*, the concentration of DHSATD and THADD together could be about 0.33 mM according to the calculations above. Unfortunately, it is unclear, in which ratio DHSATD and THADD are formed in this culture. However, the concentration of DHSATD might be something between 0.1 and 0.3 mM. As DHSATD was used in concentrations twice as high as in cultures of *P. stutzeri* Chol1 pBBR1MCS-5::*hsh2*, the concentraion of DHSATD should be approximately 0.2 – 0.6 mM. According to these calculations, the stock solution of DHSATD had a concentration of 2 – 6 mM.

Figure S6 Calculation of the approximate DHSATD (XI in Figure 1) concentration in test cultures for MDTETD (XIII) production.

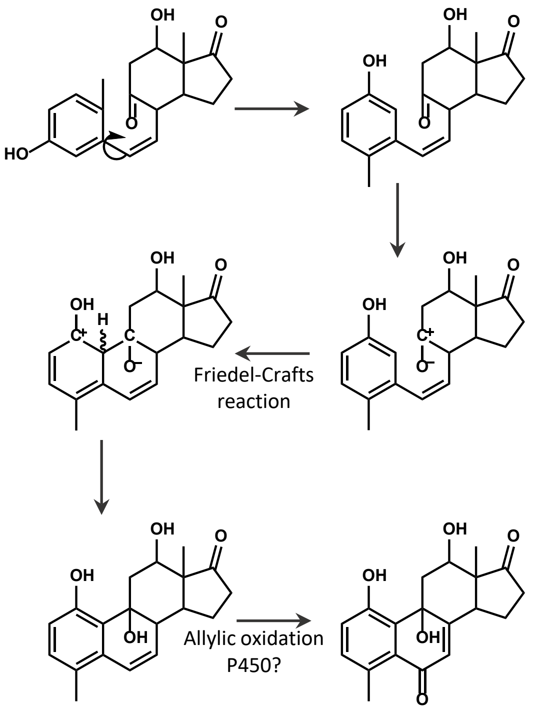

Figure S7 Proposed (bio)chemical reaction pathway from DHSATD (XI in Figure 1) to MDTETD (XIII) via the potential intermediate XIV (see Figure 6D).

References in Supplemental Material

1. Philipp B, Erdbrink H, Suter MJF, Schink B. 2006. Degradation of and sensitivity to cholate in *Pseudomonas* sp. strain Chol1. Arch Microbiol 185:192–201.
2. Yücel O, Drees S, Jagmann N, Patschkowski T, Philipp B. 2016. An unexplored pathway for degradation of cholate requires a 7α-hydroxysteroid dehydratase and contributes to a broad metabolic repertoire for the utilization of bile salts *in Novosphingobium* sp. strain Chol11. Environ Microbiol 18:5187–5203.
